# Characterization of the genomes of cluster CT *Gordonia terrae* phages, Horseradish and Yummy

**DOI:** 10.1128/MRA.00747-23

**Published:** 2023-11-20

**Authors:** Nicole Bazinet, Esther Biro, Ashley Geydoshek, Grace Hodgkin, Eric Jestel, Emily Klute, Clare MacDonald, Marcus Russano, Jayson Thatcher, Melody N. Neely, Sally Molloy

**Affiliations:** 1 The Honors College, University of Maine, Orono, Maine, USA; 2 Department of Molecular and Biomedical Sciences, University of Maine, Orono, Maine, USA; 3 School of Biology and Ecology, University of Maine, Orono, Maine, USA; Department of Biology, Queens College, Queens, New York, USA

**Keywords:** bacteriophage, Actinobacteria, genome

## Abstract

The genomes of lytic, cluster CT *Gordonia terrae* phages, Horseradish and Yummy, are 45,764 and 45,878 bp in length, respectively, and each encodes 71 protein-coding genes. The genomes are identical in sequence with the exception of a 38-bp insertion/deletion in the minor tail protein, gp26.

## ANNOUNCEMENT

**Fig 1:**
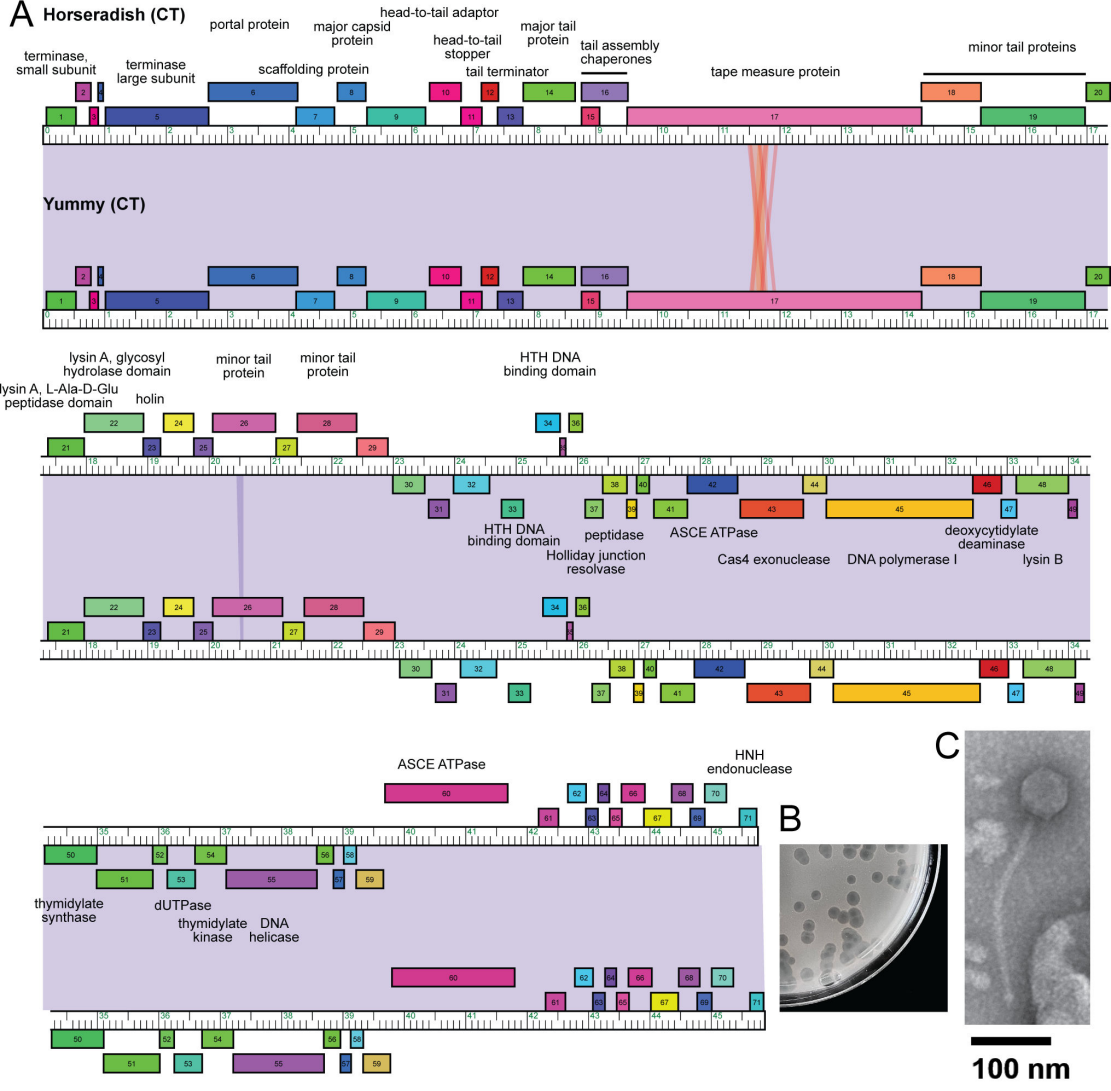
(**A**) Genome map of *Gordonia* phages Horseradish and Yummy. The genome coordinates are represented by the ruler in units of kilobase pairs. The colored boxes above and below the ruler represent genes transcribed in the forward and reverse directions, respectively. Genes were assigned to a phamily using Phamerator in the Actino_draft database, and different phamilies are indicated by colors (4). Shading between the genomes indicates nucleotide identity with violet being the most similar. Predicted functions are centered above or below forward and reverse-transcribed genes, respectively. (**B**) The plaque morphology of Horseradish on a lawn of *G. terrae*. (**C**) An electron micrograph of Horseradish is shown in the inset with a scale bar of 100 nm.

Actinobacteriophages are diverse and abundant viruses that infect bacteria within the phylum Actinobacteria (1). Programs such as Science Education Alliance-Phage Hunters Advancing Genomics and Evolutionary Science (SEA-PHAGES) enable students to expand their understanding of viral genomes and their interactions with bacteria (2). Actinobacteriophages Horseradish and Yummy were isolated from soils collected on 2 September 2022 in Old Town, Maine (44.928847°N, 68.67177°W) and from Fay Hyland Botanical Garden in Orono, Maine (44.89550°N, 68.67546°W), respectively, on the host *Gordonia terrae* 3612. Soil extracts were prepared in peptone-yeast extract-calcium (PYCa) media and filtered on 0.22 µm filters before inoculating with *G. terrae*. After incubating at 30°C for 2 days, extracts were diluted and plated in soft agar containing *G. terrae* onto PYCa agar. Horseradish and Yummy plaques were purified by four rounds of plaque assays, and each forms clear, 3.5 mm plaques on a lawn of *G. terrae* after 2 days of incubation at 30°C (3). Neither Yummy nor Horseradish form stable lysogens of *G. terrae* (3). The phage lysates were examined by negative-stained transmission electron microscopy revealing *Siphoviridae* morphologies (Fig. 1). Horseradish particles have long, flexible, noncontractile tails 290.2 ± 5.64 nm (mean ± standard error ) in length and an icosahedral head 64.7 ± 0.98 nm in diameter (*n* = 7). Yummy particles are slightly smaller with 245.6 ± 7.05 nm tails and icosahedral heads 56.4 ± 0.94 nm in diameter (*n* = 5).

A DNA phenol-chloroform extraction method was used on high-titer lysates before DNA was prepared for sequencing with the NEBNext Ultra II library preparation kit (New England BioLabs, Ipswich, MA, USA) (5). An Illumina MiSeq platform produced 130,000 and 673,600 single-end 150-bp reads for the Horseradish and Yummy genomes, respectively. Newbler v2.9 and Consed v29 were used for *de novo* assembly and checks for completeness yielding a 45,764-bp Horseradish genome and a 45,878-bp Yummy genome with shotgun coverages of 1,038-fold and 201-fold, respectively (6). Genome ends are defined by single-stranded 10-bp 3′ sticky overhangs (CGGTAGGCTT) (7). The Horseradish and Yummy genomes share >35% gene content with members of cluster CT in the Phamerator Actino_Draft (version 521) database and were assigned to this cluster (Fig. 1) (4, 8, 9).

Using DNA Master v5.23.6 (http://cobamide2.bio.pitt.edu/) and PECAAN (https://blog.kbrinsgd.org/), the genomes were autoannotated with GeneMark V2.5 and GLIMMER v3.02b (10, 11). Translational starts were manually chosen based on the inclusion of coding potential predicted with genemark.hmm and conservation across homologs using BLAST and Starterator analyses (10, 12). BLAST, TMHMM, and HHpred were used to predict putative gene functions (12–14). In each genome, 71 protein-coding genes were detected, and no tRNA genes were identified by ARAGORN v1.2.38 or tRNAscan-SE v2.0 (15, 16). Phamerator was used to prepare genome maps in the Actino_Draft database (4).

The genomes are identical in sequence with the exception of a 38-bp indel in gp26. The left arms contain (gp1–gp28) forward-transcribed structural and assembly genes (Fig. 1). The lysis cassette is located within the minor tail proteins and includes holin (gp23) and the L-Ala-D-Glu peptidase (gp21) and glycosyl hydrolase domains of lysin A. Lysin B gene (gp48) is located on the right arm of the genomes. The right arms contain forward- and reverse-transcribed genes with DNA replication and metabolism functions including DNA polymerase I (gp45) and DNA helicase (gp55).

## Data Availability

Horseradish is available at GenBank with accession no. OR159672 and the Sequence Read Archive (SRA) no. SRX20165766. Yummy is available at GenBank with accession no. OR159652 and the Sequence Read Archive (SRA) no. SRX20165785.
